# Intrathecal Delivery of Mesenchymal Stromal Cells Protects the Structure of Altered Perineuronal Nets in SOD1 Rats and Amends the Course of ALS

**DOI:** 10.1002/stem.1812

**Published:** 2014-11-26

**Authors:** Serhiy Forostyak, Ales Homola, Karolina Turnovcova, Pavel Svitil, Pavla Jendelova, Eva Sykova

**Affiliations:** a Institute of Experimental Medicine, Academy of Science of the Czech RepublicPrague, Czech Republic; b Department of Neuroscience, 2nd Medical Faculty, Charles UniversityPrague, Czech Republic

**Keywords:** Neurodegeneration, Stem cells, Extracellular matrix, Proteoglycans, Preclinical trials

## Abstract

Amyotrophic lateral sclerosis (ALS) is a progressive neurodegenerative disorder resulting in a lethal outcome. We studied changes in ventral horn perineuronal nets (PNNs) of superoxide dismutase 1 (SOD1) rats during the normal disease course and after the intrathecal application (5 × 10^5^ cells) of human bone marrow mesenchymal stromal cells (MSCs) postsymptom manifestation. We found that MSCs ameliorated disease progression, significantly improved motor activity, and prolonged survival. For the first time, we report that SOD1 rats have an abnormal disorganized PNN structure around the spinal motoneurons and give different expression profiles of chondroitin sulfate proteoglycans (CSPGs), such as versican, aggrecan, and phosphacan, but not link protein-1. Additionally, SOD1 rats had different profiles for CSPG gene expression (Versican, Hapln1, Neurocan, and Tenascin-R), whereas Aggrecan and Brevican profiles remained unchanged. The application of MSCs preserved PNN structure, accompanied by better survival of motorneurons. We measured the concentration of cytokines (IL-1α, MCP-1, TNF-α, GM-CSF, IL-4, and IFN-γ) in the rats' cerebrospinal fluid and found significantly higher concentrations of IL-1α and MCP-1. Our results show that PNN and cytokine homeostasis are altered in the SOD1 rat model of ALS. These changes could potentially serve as biological markers for the diagnosis, assessment of treatment efficacy, and prognosis of ALS. We also show that the administration of human MSCs is a safe procedure that delays the loss of motor function and increases the overall survival of symptomatic ALS animals, by remodeling the recipients' pattern of gene expression and having neuroprotective and immunomodulatory effects. Stem Cells *2014;32:3163–3172*

## Introduction

Amyotrophic lateral sclerosis (ALS) is a progressive neurodegenerative disorder that causes the dysfunction of motoneurons (MNs), resulting in the sufferer's death within 3–5 years after onset. Most patients are diagnosed with ALS 10–15 months after the first symptoms appear due to the absence of specific diagnostic markers. At present, the only drug of choice (Riluzole) does not counteract the progression of the disease, and therefore the results of such therapy are still unsatisfactory 1. New perspectives for the treatment of neurodegenerative diseases such as ALS have been opened by the discovery of stem cells (SC). Various SC types isolated from fetal or embryonic tissues have been successfully used to treat CNS in vivo and even clinical trials involving patients have been conducted 2–6. The therapeutic effects of SCs are based on their ability to secrete growth factors, generate replacements for affected or missing cells (including neurons), differentiate into neural cells, and establish functional connections between grafted and host cells after transplantation 7–10. The delivery of embryonic and induced pluripotent stem cells has been shown to promote functional, behavioral, and morphological improvements, extend survival, and even structurally integrate into the segmental motor circuitry of mutant superoxide dismutase 1 (SOD1) animals 11–15.

Adult stem cells, such as bone marrow mesenchymal stromal cells (MSCs), isolated from mature organisms, could be used as an alternative to embryonic and fetal cells with a smaller risk of side effects. All MSC populations (bone marrow, adipose tissue, etc.) have been shown in in vitro studies to express a large variety of neuronal genes and transcription factors with a wide differentiation potential 16–18. The therapeutic effects of MSCs are very complex, but could be explained by the secretion of a wide range of substances, either by host cells or by the MSCs themselves (paracrine function), that play a crucial role in nourishing neurons, reducing neuronal sensitivity to glutamate receptor ligands, altering gene expression, and thus reactivating cell plasticity in the CNS 19. In vitro, in vivo, preclinical, and clinical safety studies have shown that the generation and grafting of MSCs effectively enhances repair, slows apoptosis, and supports the remaining host MN, inhibits inflammation, and does not induce an immune response or tumor formation 20–24. Moreover, MSCs are also known for transferring and propagating functional mitochondria into the target cells, thus rescuing the host cells' mitochondrial functions 25,26. The above properties of MSCs make these cells among the leading candidates for application in patients 19.

Normal functioning of the CNS depends not only on the interaction of neural cells with other cell types but also on a healthy extracellular matrix (ECM). The perineuronal net (PNN) is a layer of condensed pericellular matrix that aggregates and wraps around the soma and proximal dendrites of interneurons and MN 27. PNNs are made of a hyaluronan backbone, to which several types of chondroitin sulfate proteoglycans (CSPGs) are bound through cartilage link protein-1 and with tenascin-C and tenascin-R molecules binding to the core proteins 28–30. PNNs can be visualized starting from the late period of development (critical period), when ECM molecules form stable aggregates triggered by the upregulation of link protein-1 29. ECM is involved in the formation of synaptic connections as well as in CNS plasticity. For example, CSPGs have been shown to play an important role in axonal guidance during CNS development and regeneration 27,31,32. Conversely, CSPGs are upregulated in activated astrocytes after injury to the CNS, leading to the restriction of anatomical and synaptic plasticity 33,34. Aberrant PNN formation has been reported to contribute to the onset of schizophrenia 35. Accumulation of some CSPGs (neurocan and versican) in the ventral spinal cord has been related to neurodegeneration in the rat model of ALS 36. Matrix metalloproteinase-9 (MMP-9), that belongs to a large family of extracellular proteases, has been shown to be altered in ALS-patients and to define and trigger the degeneration of MN subsets that are destined to die 37,38. Impairment of ECM structures and molecules might have important implications for the understanding of pathogenesis, development of novel strategies for diagnosis, and early treatment of CNS disorders. However, there is still a lack of information on this topic. The aim of this study is to investigate mRNA and protein expression of CSPGs (versican, aggrecan, phosphacan, and link protein-1), as well as to evaluate the effect of intrathecal delivery of human bone marrow MSCs on the PNN structure and the course of the disease, using symptomatic SOD1-transgenic rats.

## Materials and Methods

An extended Materials and Methods section is available as Supporting Information (Supporting Information Methods).

## Results

### MSCs Characteristics

Human MSCs (hMSCs) were characterized according to the recommendations of the International Society for Cellular Therapy 39. Tested hMSCs expressed the following mesenchymal surface markers: CD29, CD44, CD73, CD90, and CD105; meanwhile, the cells were negative for CD34, CD45, CD235a, and CD271 (Supporting Information Fig. S1A). MSCs used in the study differentiated toward three phenotypes: osteogenic, adipogenic, and chondrogenic, as proven by staining for calcium deposits (Alizarin red), oil droplets (oil red O), and acid mucopolysaccharides (Alcian blue), respectively (Supporting Information Fig. S1B–S1D). Cultured MSCs expressed a major cytoskeletal component of mesenchymal cells, vimentin (Supporting Information Fig. S1E).

### Effects of MSC Implantation on Rat Motor Function and Survival

**Figure 1:**
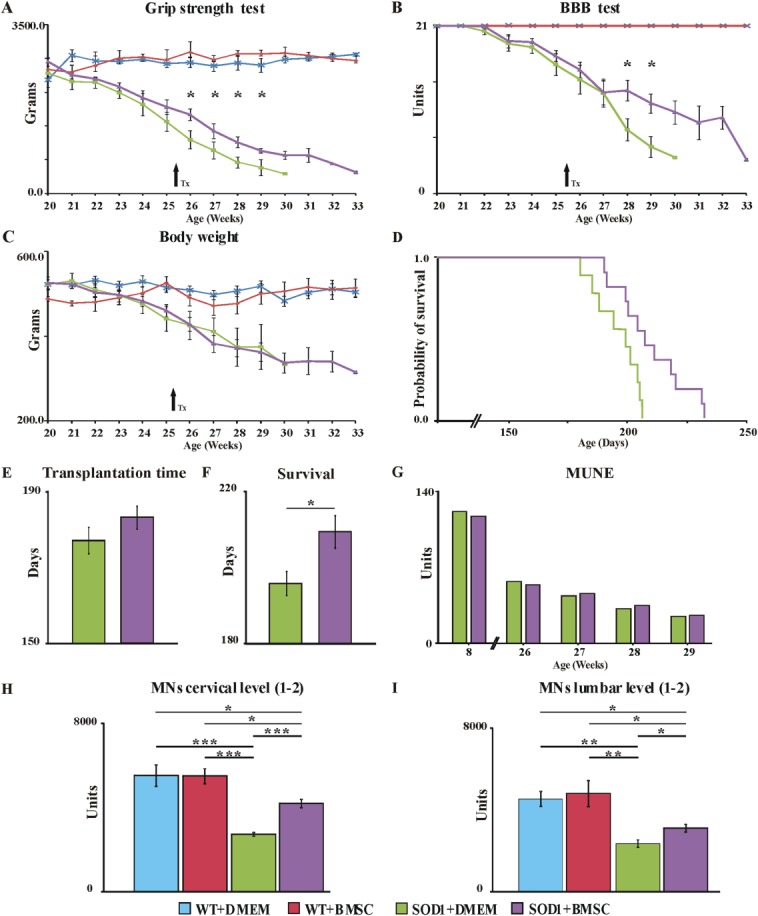
Delivery of bone marrow mesenchymal stromal cells (BMSC) significantly extended the disease course and lifespan and supported MN survival when compared with vehicle-injected animals. Grafted MSCs significantly slowed the decline in limb grip strength (A) and motor performance of SOD1 rats (B), but did not affect the body weight loss (C). The Kaplan-Meier method shows significantly better survival of MSC-grafted SOD1 rats (D, F). The beginning of the disease did not differ between the two groups of SOD1 rats (E). MUNE in the medial gastrocnemius muscle showed no difference in motor unit numbers between MSC-treated and vehicle-injected SOD rats (G). The combination of IHC staining with an unbiased stereological method on serial sections of the spinal cord showed a significantly higher number of MN in MSC-treated animals at the cervical (H) and lumbar (I) levels. Arrows indicate the time of transplantation. Error bars indicate SEM. Significance at: *, *p* ≤ .05; **, *p* ≤ .01; ***, *p* ≤ .001. Abbreviations: BBB, Basso-Beattie-Bresnahan test; DMEM, Dulbecco's modified Eagle's medium; MN, motoneurons; MUNE, motor unit number estimation; SOD1, superoxide dismutase 1; WT, wild type.

Rats were considered for MSCs/vehicle transplantation based on the following criteria: dropped BBB score from 21 to 17–16, decreased grip strength by more than 400 g, and/or the loss in body weight (Fig. 1A, 1B). The beginning of the disease did not differ between the two groups of SOD1 rats (Fig. 1E). However, the application of MSCs significantly improved motor performance (Basso-Beattie-Bresnahan [BBB] score test, *p* ≤ .05) and muscle strength (grip strength test, *p* ≤ .05) when compared with vehicle-treated rats. The difference in motor activity between the two groups gradually increased with the course of the disease, whereas the dynamics of body weight loss did not mirror this (Fig. 1A–1C). The wild-type (WT) rats that received MSCs/Dulbecco's modified Eagle's medium (DMEM) did not show changes in tested parameters related to the cell/vehicle delivery (Fig. 1A–1C). Motor unit number estimation (MUNE) in the medial gastrocnemius muscle revealed the following baseline values of motor units number for group of MSC-treated (*n* = 6) and the vehicle-injected animals (*n* = 6): 131 ± 15, and 127 ± 18, respectively. At 26 weeks of age, a significant reduction in the number of motor units was found (64 ± 12 vs. 59 ± 10) compared to the baseline values. As the disease progressed, the number of motor units continuously declined to 26 ± 4 versus 25 ± 6 at 29 weeks, but no statistical differences between both groups of SOD1 rats were found (Fig. 1G). Motor nerve conduction velocity was derived from latencies of M waves, elicited by electrical stimulation of the sciatic nerve. Latencies of M waves at 8 weeks were 1.95 ± 0.05 ms versus 1.87 ± 0.03 ms (MSC-treated vs. vehicle-treated) and did not changed significantly during the disease course (1.88 ± 0.02 ms vs. 1.91 ± 0.03 ms at 29 weeks). However, transplantation of 5 × 10^5^ MSCs was sufficient to significantly extend the lifespan of the cell-treated rats (group mean 209.3 and 195.7 days, respectively; *p* ≤ .05) by almost 14 days (Fig. 1D, 1F).

### Neuroprotective Effect of MSCs on MN

SOD1 rats, regardless of experimental treatment, showed significantly lower numbers of cervical (Fig. 1H) and lumbar (Fig. 1I) MN, compared to age-matched WT rats. However, the delivery of MSCs into symptomatic SOD1 rats partially rescued cervical (*p* ≤ .001) and lumbar (*p* ≤ .05) MN. No differences between the MSCs- and vehicle-treated groups were detected using MUNE analysis; however, the significantly better survival of MN in the spinal sections of MSC-treated rats favors the dying-back axonopathy theory of ALS development, when MN are partially rescued by MSCs, but their axons are unable to properly innervate the target muscles.

### Survival and Biodistribution of MSCs

To determine the survival and fate of the grafted hMSCs, spinal cord sections were stained with human-specific markers for mitochondria (MtC02) and nuclei (HuNu). Despite the triple immunosuppression of the animals, we did not find cells positively stained with human-specific antibodies in the longitudinal sections. Therefore, in parallel we performed experiments using rat MSC^GFP+^, that were intrathecally delivered following the same protocol. Two weeks later, animals were sacrificed and their organs were used for histological evaluation of cell survival and biodistribution. Similarly to hMSCs, 14 days after cell transplantation samples were analyzed and no green fluorescent protein (GFP)-positive cells were discovered in either the cerebrospinal fluid (CSF) or histological sections of the spinal cord, brain, lungs, spleen, or liver.

### Changes to PNNs

**Figure 2:**
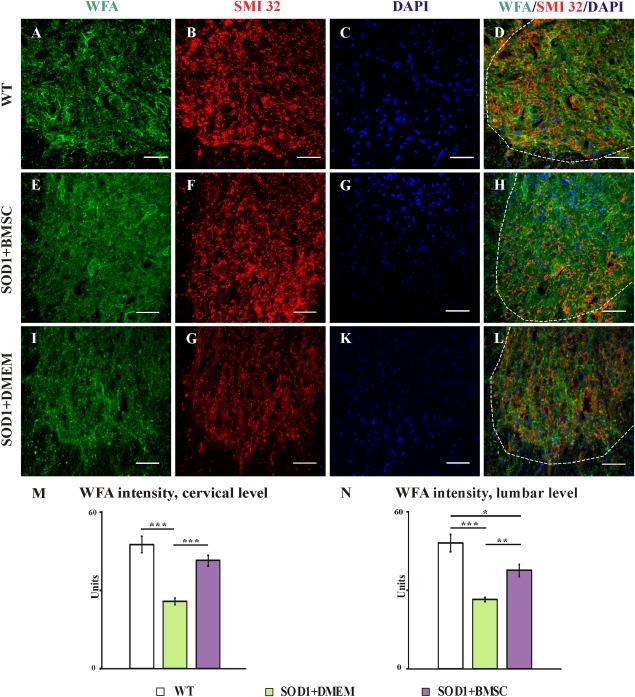
WFA staining visualizes the perineuronal nets (PNNs) around motoneurons (SMI 32) in the ventral horns of wild-type (A–D), end-stage SOD1 mesenchymal stromal cell-treated (E–H) and symptomatic SOD1 sham-treated (I–L) rats. A further quantification of WFA staining intensity from the ventral horns (interrupted line) of MSC-treated SOD1 rats (violet columns) showed that these animals had a significantly better preserved PNNs in both the cervical (M) and lumbar (N) levels of the spinal cord, when compared with sham-treated SOD1 animals (green columns) and age-matched WT littermates (empty columns). Both groups of SOD1 rats had attenuated PNNs; however, this difference was not significant at the cervical level, where the MSCs were delivered, whereas at the lumbar level all SOD1 rats had a significantly weaker intensity of PNN staining (M, N). Error bars indicate SEM. Significance at: *, *p* ≤ .05; **, *p* ≤ .01; ***, *p* ≤ .001 (scale bars = 50 µm). Abbreviations: BMSC, bone marrow mesenchymal stromal cells; DMEM, Dulbecco's modified Eagle's medium; SOD1, superoxide dismutase 1; WFA, Wisteria floribunda agglutinin; WT, wild type.

By evaluating samples stained with immunohistochemistry (IHC), we found that WT animals have normal PNNs around MNs (Fig. 2A–2D), whereas all transgenic (Tg) rats at the end-stage of the disease had disorganized and attenuated PNN structures (Fig. 2E–2L). Interestingly, MSC-treated SOD1 rats (Fig. 2E–2H), despite having attenuated PNNs when compared with WT animals, displayed a significantly reduced deterioration of their PNN structures when compared with vehicle-treated rats (Fig. 2I–2L). Quantification of Wisteria floribunda agglutinin (WFA) staining intensity showed that all SOD1 rats had a lower fluorescent intensity at the cervical (Fig. 2M) and lumbar (Fig. 2N) levels when compared with WT age-matched littermates (*p* = .108 and *p* = .012, respectively). The delivery of MSCs into symptomatic rats significantly preserved their PNNs at the cervical (Fig. 2M) and lumbar levels of the ventral horns (Fig. 2N) when compared with vehicle-injected SOD1 rats (*p* = .000008 and *p* = .00021, respectively). We found a significant difference in WFA staining intensity between the cervical and lumbar levels within the cell-treated SOD1 group (*p* = .026) and no changes in the WT and vehicle-injected groups, suggesting that proximity to the site of cell application is essential to achieve the best effects implied.

**Figure 3:**
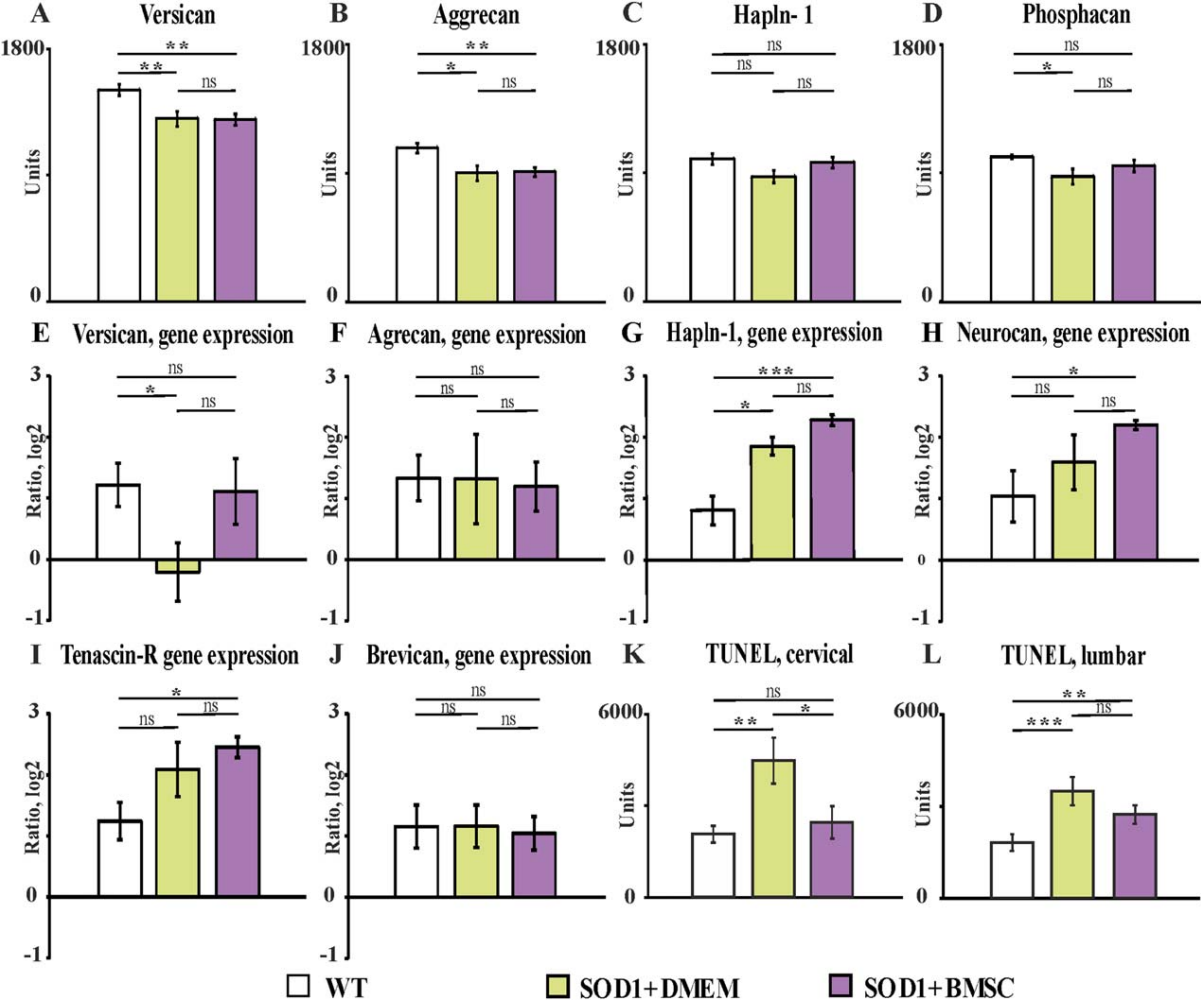
Semiquantitative analysis of chondroitin sulfate proteoglycans (CSPGs) immunofluorescence staining intensity measured in the spinal ventral horns (A–D). Gene expression profiles of CSPGs were evaluated in the spinal cords of wild-type and SOD1 transgenic rats by means of the RT-qPCR method (E–J). The level of apoptosis in the spinal cord was studied by means of the TUNEL assay (K, L). Error bars indicate SEM. Significance at: *, *p* ≤ .05; **, *p* ≤ .01; ***, *p* ≤ .001. Abbreviations: BMSC, bone marrow mesenchymal stromal cells; DMEM, Dulbecco's modified Eagle's medium; SOD1, superoxide dismutase 1; WT, wild type.

IHC evaluation of CSPGs revealed significantly decreased expression of versican (Fig. 3A, *p* = .006 [WT vs. DMEM] and *p* = .0011 [WT vs. MSC]; Supporting Information Fig. S2) and aggrecan (Fig. 3B, *p* = .011 [WT vs. DMEM] and *p* = .0027 [WT vs. MSC]; Supporting Information Fig. S3) in the ventral horns of SOD1 rats compared with WT littermates and did not show any difference in the expression of these proteins between MSC-treated and vehicle-injected SOD1 rats. The expression of link protein-1 (Fig. 3C; Supporting Information Fig. S4) was comparable among all groups of animals (*p* = .053 [WT vs. DMEM], *p* = .16 [MSC vs. DMEM], and *p* = .7 [WT vs. MSC]). Anti-phosphacan antibody staining intensity (Fig. 3D; Supporting Information Fig. S5) was significantly decreased in the vehicle-injected SOD1 animals when compared with WT rats (*p* = .026) and did not differ from that of MSC-treated SOD1 animals.

### RT-qPCR Evaluation of CSPG Gene Expression

The expression of mRNA for the main core proteins was studied in SOD1 rats and compared with those of age-matched littermates. Versican (transcript variants, V1–4) mRNA expression in the spinal cord of sham-treated SOD1 rats was significantly decreased when compared with WT animals (*p* = .042), while the MSC-treated SOD1 rats did not show any difference (Fig. 3E). Despite significant changes in the expression of aggrecan between WT and Tg animals, there were no changes in the corresponding gene expression in the spinal cords (Fig. 3F).

In contrast, there was a significant difference in *hapln1* mRNA expression (Fig. 3G) between SOD1 (MSC- and sham-treated) and WT rats (*p* = .0007 and *p* = .015, respectively), despite the similar expression of corresponding proteins in the spinal cords of WT and SOD1 animals. Upregulation of *hapln1* mRNA expression in MSC-treated SOD1 animals when compared with vehicle-treated rats was shown to be near significance (*p* = .054). Other PNN compounds, neurocan (Fig. 3H) and tenascin-R (Fig. 3I), had upregulated mRNA expression only in MSC-treated SOD1 rats when compared with WT littermates (*p* = .039 and *p* = .014, respectively). The analysis of brevican mRNA expression was very consistent and showed no differences among the groups (Fig. 3J). At this stage, it remains unresolved whether the above changes are primary or a reflection of CNS degeneration and disease progression. Future experiments studying the timing of PNN structural changes at various stages of disease progression will be needed.

### Analyses of Apoptosis (TUNEL Assay)

The intensity of TUNEL staining measured in the ventral horns at the cervical level (Fig. 3K) was significantly higher in the vehicle-treated SOD1 rats when compared with WT (*p* = .001) and MSC-treated SOD1 rats (*p* = .01); we found no differences between MSC-treated and WT rats (*p* = .29). At the lumbar level we observed that both cell- and vehicle-treated SOD1 rats had a significantly greater degree of DNA fragmentation compared to WT littermates (*p* = .004 and *p* = .0007, respectively), but we did not observe significant changes between MSC-treated and vehicle-injected Tg subjects (Fig. 3L).

### Analyses of Cytokines in the CSF

**Figure 4:**
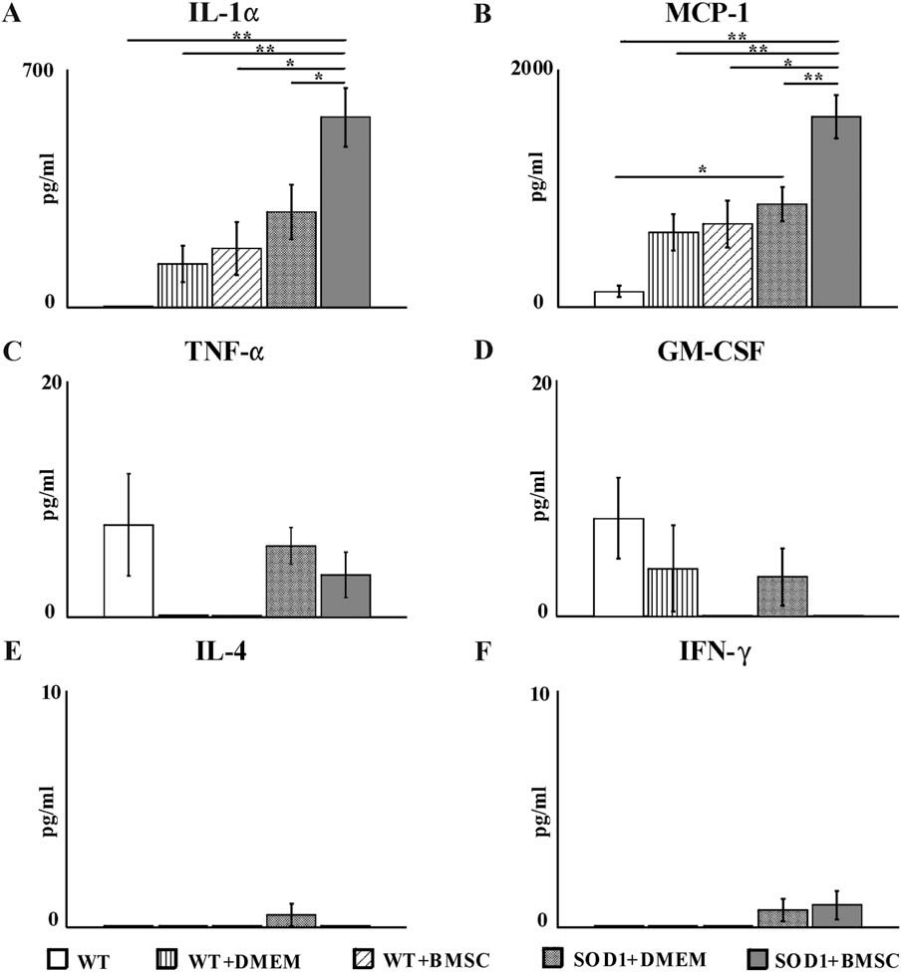
Cytokine levels IL-α (A), MCP-1 (B), TNF-α (C), GM-CSF (D), IL-g4 (E), IFN-γ (F) measured by a fluorescence-activated cell sorting-bead assay in the CSF of SOD1 and WT littermates with and without the application of mesenchymal stromal cells. Error bars indicate SEM. Significance at: *, *p* ≤ .05; **, *p* ≤ .01; ***, *p* ≤ .001. Abbreviations: BMSC, bone marrow mesenchymal stromal cells; DMEM, Dulbecco's modified Eagle's medium; SOD1, superoxide dismutase 1; WT, wild type.

The CSF of cell-treated SOD1 rats contained significantly higher concentrations of IL-1α (*p* ≤ .05) and MCP-1 (*p* ≤ .01) when compared with sham-treated SOD1 and WT rats (Fig. 4A, 4B). There was also a tendency toward an increase in TNF-α (Fig. 4C) and IFN-γ (Fig. 4F) levels in the CSF of both SOD1 groups when compared with cell- and vehicle-injected WT rats. Interestingly, neither the SOD1 nor the WT groups of MSC-treated rats showed any signs of GM-CSF in their CSF (Fig. 4D). IL-4 was either absent in the CSF or present in very small quantities in all groups of animals (Fig. 4E).

## Discussion

This study was designed as a preclinical trial aiming to evaluate the regenerative effect and safety of hMSCs delivered intrathecally into SOD1 transgenic rats after the manifestation of the first disease symptoms. The application of hMSCs significantly improved motor activity, prolonged survival, decreased apoptosis, and protected the spinal MN of SOD1 rats. Our results support the dying-back theory of ALS development. We tested the hypothesis that changes in PNN structure are involved in the pathobiology of ALS. For the first time, we report that the ECM of SOD1 animals has a different composition than that of age-matched WT littermates, as indicated by a disorganized PNN structure around the spinal MN and the different immunohistological profiles of some spinal CSPGs (versican, aggrecan, and phosphacan, but not hapln1). We showed that SOD1 rats have different gene expression profiles of some spinal CSPGs (*Versican*, *Hapln1*, and *Tenascin-R*), whereas others (*Aggrecan*, *Neurocan*, and *Brevican*) remained unchanged. Moreover, hMSC application rescued the PNN structure around spinal MNs and affected the expression of some CSPG genes, suggesting the reactivation of CNS plasticity. Finally, we found significantly higher concentrations of IL-1α and MCP-1 in the CSF of SOD1 compared to WT rats.

Different routes for the transplantation of MSCs have been tested: local (intraspinal, intramuscular), systemic (intravenous, intra-arterial, etc.), and combined, each having their advantages and disadvantages. To ensure the study can be successfully translated from the bench to the bedside, the main criterion that should be followed while designing a preclinical trial should be safety and minimal invasion of the methods used. In our previous work, we showed how the combined (intraspinal and intravenous) transplantation of rat MSCs into SOD1 animals led to a 6% increase in survival and significantly better preserved motor functions 21. In this study, we used the intrathecal route and found a significantly prolonged lifespan (by 7%), higher motor activity and increased MN survival. We believe that access to epidural space via lumbar puncture and intrathecal drug application is a clinically relevant, noninvasive, and safe procedure that is already widely used in practice, and could therefore be effectively used for cellular therapy of patients with ALS. Moreover, if necessary this procedure could be repeated enabling repeated cell application. Our results are consistent with those of Boucherie et al., who reported the prolongation of lifespan and MN survival after intrathecal delivery of rat MSCs into symptomatic SOD1 animals 40; however, our results might be more clinically relevant considering the type and number of the grafted cells. A similar study by Morita et al. reported delayed disease onset and better survival of MSC-treated females, but not males, after grafting rat MSCs onto the fourth ventricle 41. Some studies, including those already mentioned by Morita and Boucherie, describe the abundant survival, migration, and astroglial differentiation of grafted MSCs, whereas others show limited viability and migration of hMSCs 42,43 or do not report graft survival at all 44. Our observations indicate that despite adequate immunosuppression, the transplants did not survive in the CNS or subdural space in either WT or SOD1 rats. We asked whether the MSCs did not survive due to their heterogenic origin, an insufficient number of transplanted cells or perhaps because of the CSF composition. To answer these questions, we performed experiments in which the same number of rat MSCs^GFP+^ was delivered into immunosuppressed age-matched WT rats. Surprisingly, already 14 days after cell transplantation GFP-positive cells could not be detected in the spinal cord, brain, lungs, spleen, or liver. We can speculate that the MSCs did not survive due to the extraneous environment and/or the composition of the CSF, rather than the cells' origin. Nevertheless, the time spent by the transplant in the host organism seems to be sufficient to potentiate better MN survival, exert an antiapoptotic effect, and extend lifespan. Moreover, proximity to the delivery site results in various neuroprotective effects: more distal equals less effective. We can assume that the long-term survival of the graft is not crucial to achieve the protective potential of MSCs, and it is probable that repeated implantation(s) of MSCs could potentially lead to an even better outcome. On the platform of current and similar studies from other groups, a 3-year, nonrandomized, open label clinical trial was launched in March 2012, in Prague (Czech Republic), aiming to assess the safety and efficacy of autologous multipotent MSCs applied to patients with a confirmed diagnosis of ALS (http://www.sukl.eu). So far, 20 patients have been recruited for the trial and injected with autologous MSCs via lumbar puncture without any adverse effects 19.

Considering that spinal PNNs form around MNs and that ALS is characterized by the selective death of MNs, we questioned the involvement of the ECM in ALS pathobiology. Using the SOD1 model of the disease we found a disorganized and vestigial spinal PNN structure in the ventral horns of terminal Tg rats; meanwhile, 1-month-old presymptomatic SOD1 rats and their WT littermates had a typical PNN pattern around their spinal MN. Earlier, Galtrey et al. identified molecules of the ECM (hyaluronan, link protein-1, aggrecan, tenascin-R, and phosphacan) that are essential for PNN formation, while others have been shown to be optional for the formation of PNNs 45. Based on these data, we analyzed the essential CSPG molecules and the corresponding gene expression profiles in SOD1 spinal cord. We found decreased expression levels of aggrecan, phosphacan, and versican, but normal link protein-1, at the terminal phase of the disease. These findings are slightly different to those published by Mizuno et al., who reported an upregulation of neurocan, versican, and phosphacan proteins in the spinal cord of the His46Arg model of ALS and related this upregulation to reactive astrogliosis 36. Unlike Mizuno, we visualized the whole PNN structure together with its core components and colocalized them with the CSPG gene expression profiles. Our results demonstrate that only the link protein-1 gene (*Hapln1*) is upregulated, while *Versican* is downregulated at the terminal stage; other genes—*Aggrecan*, *Tenascin-R*, *Neurocan*, and *Brevican*—are expressed in a similar pattern as in age-matched WT controls. The described “cross” (upregulation of the *Hapln1* and downregulation of the *Versican* genes) in the spinal cord of SOD1 rats is reminiscent of the effect described after the application of chondroitinase ABC to promote sprouting and axonal regeneration after spinal cord injury and may be a compensatory mechanism to restore the normal function of the CNS 46. This compensatory mechanism aims to reactivate plasticity, preserve the remaining neurons, and facilitate the formation of new synaptic connections. Despite the disorganized PNNs structure shown by WFA staining at the terminal stage of MSC-treated rats, CSPG analysis showed no major differences in the amount of these proteins between sham- and cell-treated transgenic animals. It is still unclear whether the changes in PNNs structure are primary or are just a reflection of neural dysfunction and neuronal death. It would also be useful to understand which lacking or dysfunctional molecules lead to PNN disintegration. The answer to these questions could unveil new mechanisms underlying ALS development, thus leading to the discovery of diagnostic markers and even effective therapies.

Under normal conditions, the spinal cord is predisposed for spontaneous neurogenesis by the mobilization of its own neural stem/progenitor cells 47,48. However, SOD1 transgenic animals are known to have limited endogenous neurogenesis and an inhospitable microenvironment 49. Considering that intrinsic neurogenesis and the renewal of ECM homeostasis are not sufficient to restore normal CNS function after the onset of ALS, we can assume that extrinsic assistance might have a beneficial effect to support the normal function and structure of CNS components. MSCs might be an ideal candidate for this purpose, as they have been shown to reduce neuronal sensitivity to glutamate receptor ligands and to alter gene expression in the damaged CNS, thus suggesting a link between the therapeutic effects of MSCs and the activation of cell plasticity 50. MSCs are also known to induce graft-to-host exchange of trophic factors, modulate innate and adaptive immune responses, stimulate angiogenesis, promote the activation, proliferation, migration, and differentiation of endogenous stem cells, and even transfer mitochondria to host cells 51,52. We could not find evidence of mitochondrial transfer from hMSCs into host cells in our experiments by means of immunohistological markers (negative human MTC02 staining). We asked if the application of MSCs could potentiate extracellular plasticity in order to maintain the PNN structure. Our results indicate that MSC-treated SOD1 rats had significantly upregulated expression of the *Hapln1*, *Neurocan*, and *Tenascin-R* genes, compared with WT controls or vehicle-injected littermates. Link protein-1 is a molecular trigger of PNN formation during the critical period and together with other CSPGs (versican or tenascin-C and -R) plays a fundamental role in limiting CNS plasticity, axonal migration, and regeneration following spinal cord injury. Based on a comparison of our data with those described during the critical period of PNN formation we could hypothesize that MSC application reactivated adult CNS plasticity 29. Additionally, PNNs have been shown to protect neurons against oxidative stress, modulate short-term glutamate receptor mobility and signal transmission as well as being involved in stem cell differentiation 29,53–55. Thus, we can assume that MSC application has a neuroprotective effect not only via a direct influence on the grafted cells to the host MN but also through the indirect preservation of the PNN structure. While the exact mechanism of plasticity reactivation is still not known one possible explanation might be the formation of exosomes by MSCs, that contain a great variety of biologically active molecules, lipids, proteins, growth-factor receptors, messenger, and microRNA, which are released into the ECM and then fused and incorporated into the host cells 19,56–58. Matrix metalloproteinases (MMPs) and their tissue inhibitor alterations have also been shown to act as fundamental effectors of ECM remodeling and stem cell mobilization 38. Thus, another possible explanation could be that after MSCs application the structure of PNN proteins was modified by MMPs (or the other way around). Future research should be focused on this topic.

The onset and progression of ALS have also been recently linked with mutant-SOD1-mediated toxicity within microglia cells, together with the activation of astrocytes and macrophages 59–61. Microglia act as a primary mediator of immune/inflammatory responses present in ALS, even before the first signs of motor dysfunction 62–64. Aggregated SOD-1 proteins stimulate macrophages and microglia to produce cytokines and chemokines (TNF-α, IL-1α, IL-1β, IL-6, GM-CSF, IL-17A, etc.), causing a suppression of the patient's T-regulatory pathways and increases their vulnerability to IL-17A-mediated damage 65. This cascade, however, could be modified by the reconstitution of T cells following bone marrow transplantation into transgenic SOD1 mice lacking functional T and B cells, resulting in the prolongation of survival 66. Extended survival of rats after intrathecal hMSC application was accompanied by a decreased level of spinal cord apoptosis and a tendency toward an elevation of IL-1α and MCP-1, accompanied by a slight decline of TNF-α in the CSF of SOD1 rats. Observed attenuated apoptosis may be explained by the cleavage of MMP3 to TNFα family ligands (FasL and TNFα, respectively) 67. Elevated MCP-1 (most likely astrocytic), which is known to have a direct neuroprotective effect in excitotoxicity and to be neuroprotective by upregulation of the neurotrophic molecule expression in astrocytes, could serve as evidence of the neuroprotective and antiapoptotic effect stimulated by intrathecal delivery of MSCs 68,69. The above chemokines are expressed at a high level even before initial ALS symptoms appear and, therefore, could potentially be used as biological markers and potential therapeutic targets 65,70.

## Conclusions

Our preclinical study demonstrates that the intrathecal administration of hMSCs is a safe procedure that is able to delay the decline in motor function and increase survival of symptomatic ALS animals. We have shown that hMSCs have a neuroprotective effect and are able to remodel the recipient's gene expression profile, thus reactivating CNS plasticity. Our study provides evidences that ECM and cytokine homeostasis are affected in SOD1 rats and that the described changes of PNN structure and the levels of MCP-1 and IL-1α in the CSF could potentially serve as biological markers for the diagnosis and prognosis of ALS. Our study has uncovered new links in the pathology of ALS, providing an optimistic future for MSC-based therapy and encourages the discovery of biological (diagnostic and prognostic) ALS markers that could be used in patients.

## Supporting Information

See www.StemCells.com for supporting information available online.
