# Supplementary figures and images for: Intrathecal Delivery of Mesenchymal Stromal Cells Protects the Structure of Altered Perineuronal Nets in SOD1 Rats and Amends the Course of ALS
Source: Stem Cells. 2014 Nov 26;32(12):3163–72. doi: 10.1002/stem.1812 (PMC4321196; doi:10.1002/stem.1812)

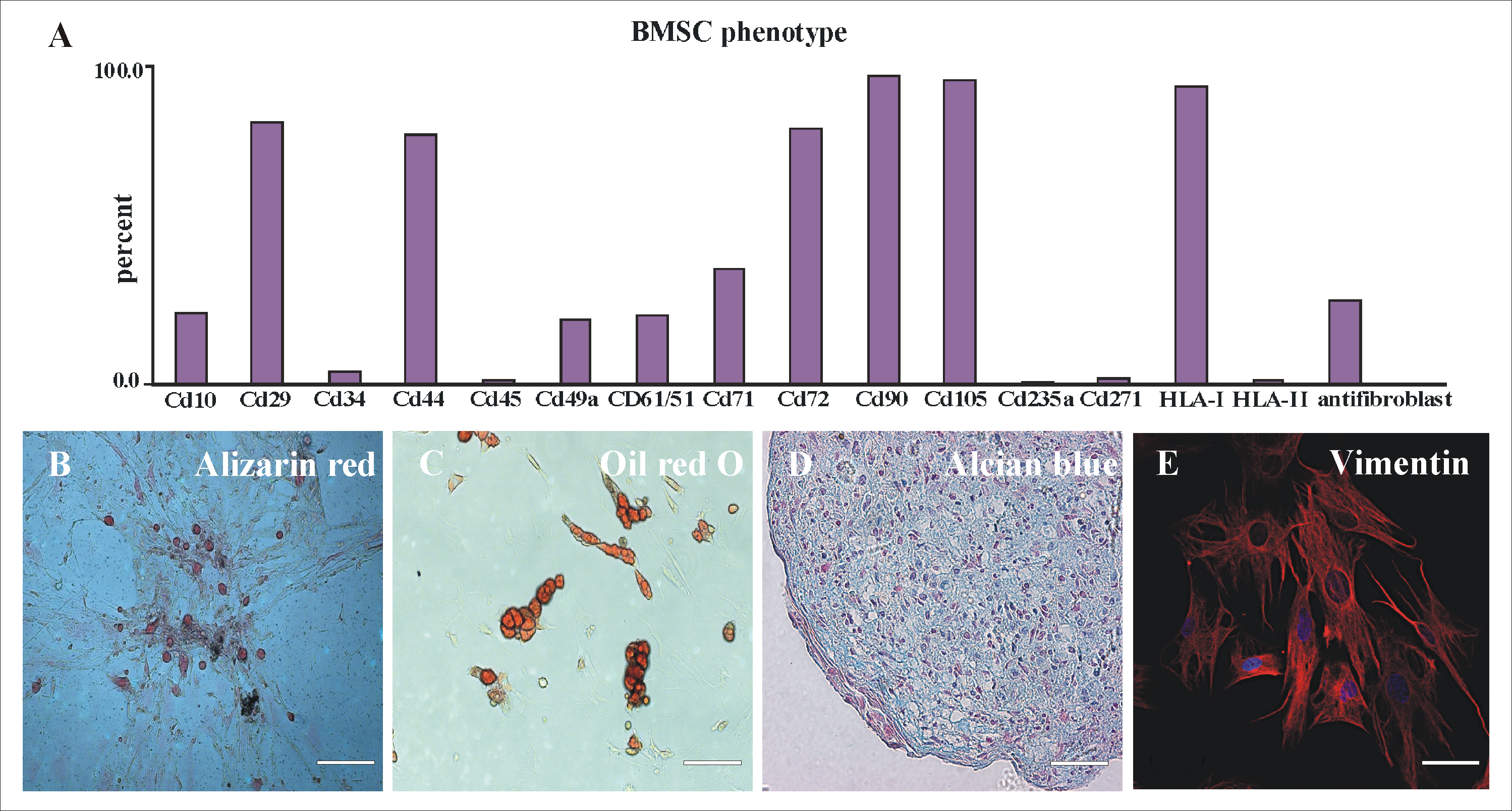

Supplement: Supplementary file 1 — Supplementary [file stem0032-3163-SD1.tif]

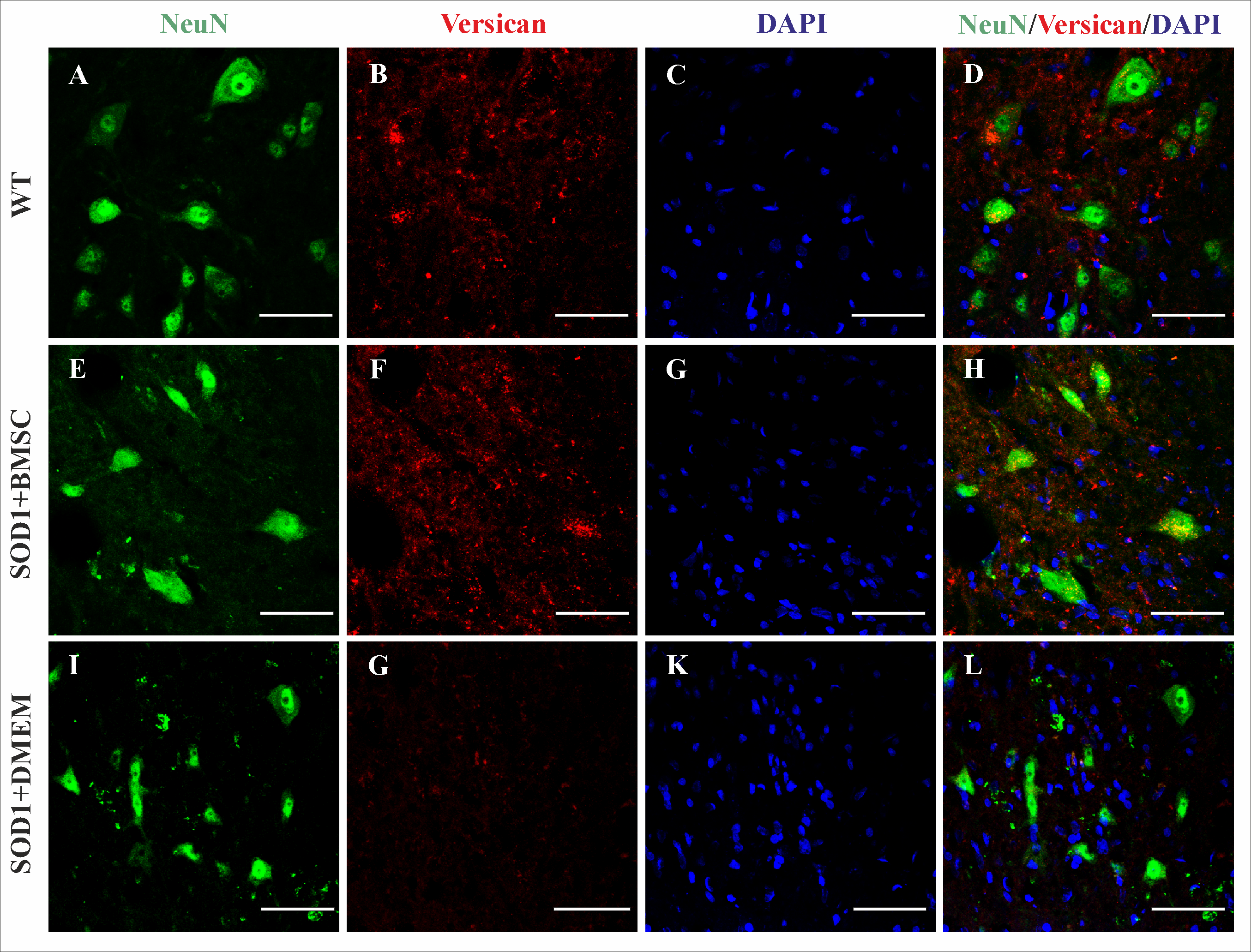

Supplement: Supplementary file 2 — Supplementary [file stem0032-3163-SD2.tif]

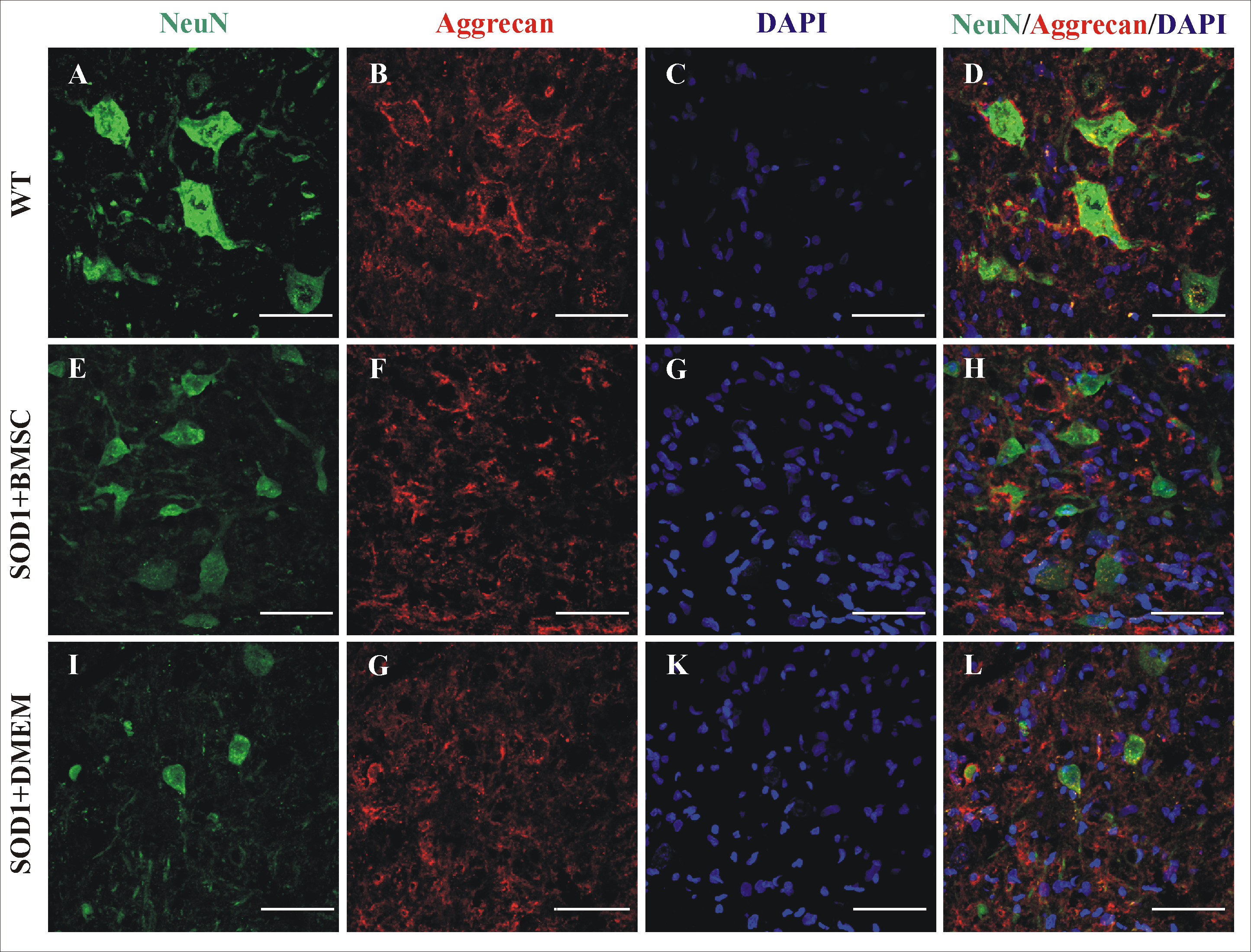

Supplement: Supplementary file 3 — Supplementary [file stem0032-3163-SD3.tif]

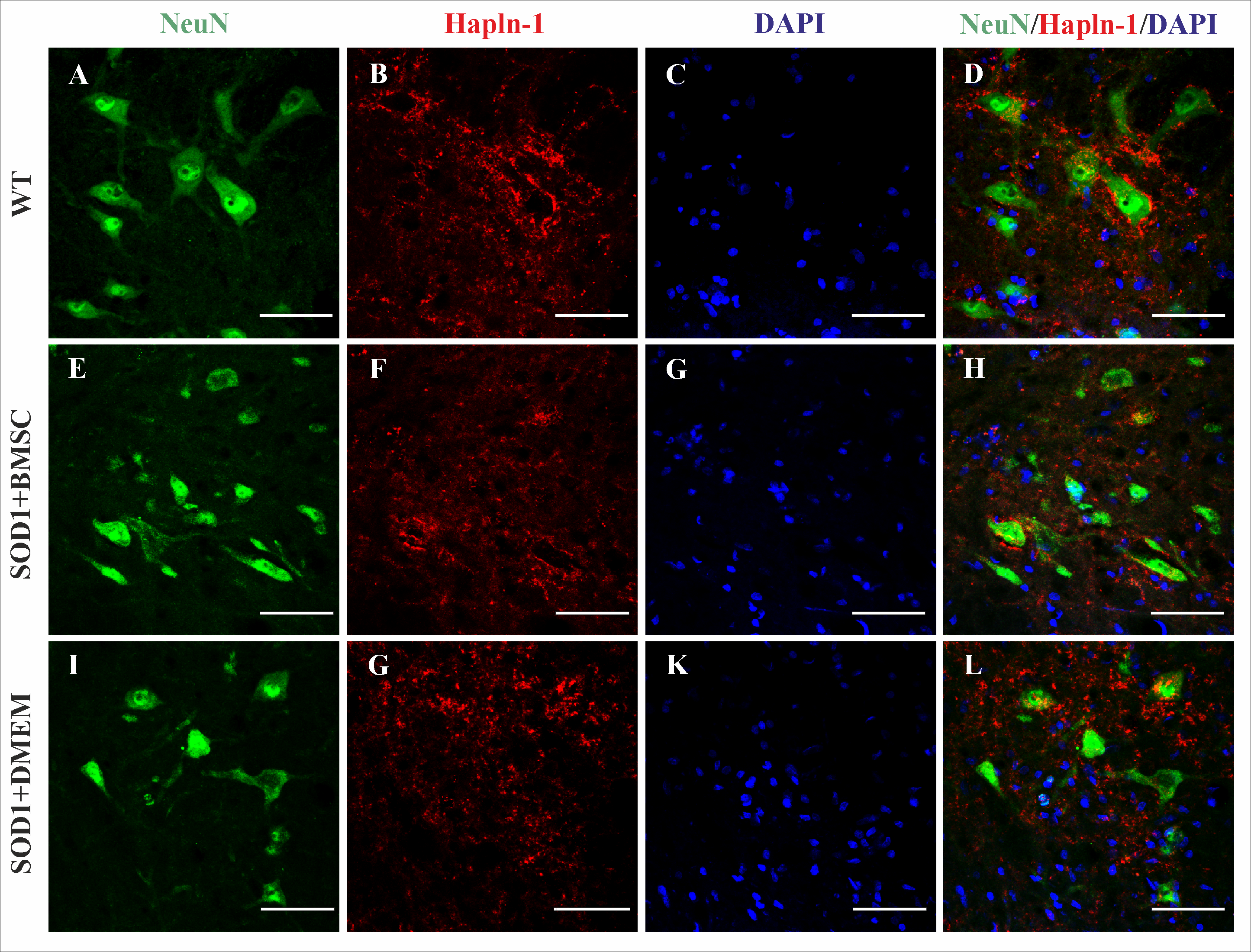

Supplement: Supplementary file 4 — Supplementary [file stem0032-3163-SD4.tif]

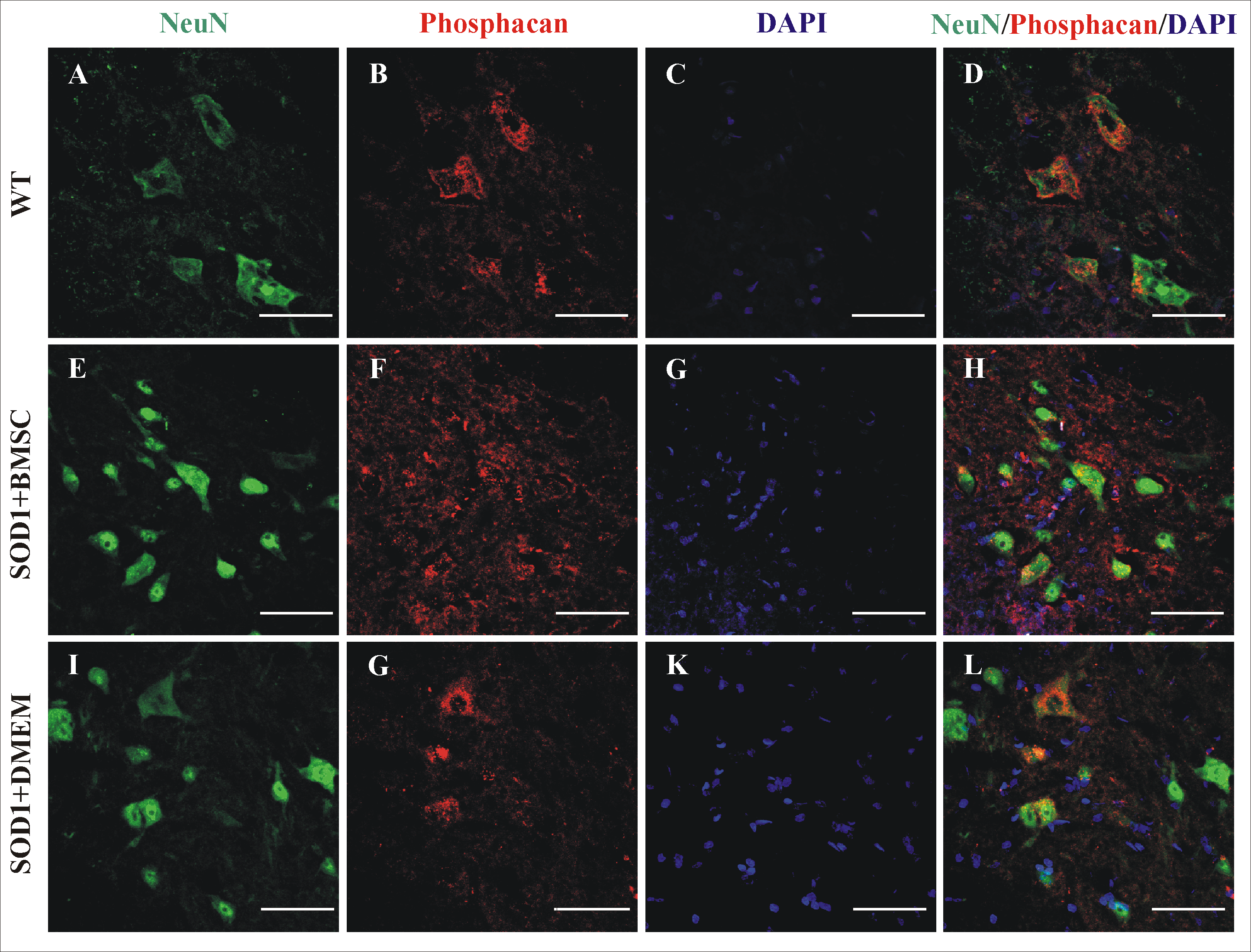

Supplement: Supplementary file 5 — Supplementary [file stem0032-3163-SD5.tif]
