# Supplementary material for: Intrathecal Delivery of Mesenchymal Stromal Cells Protects the Structure of Altered Perineuronal Nets in SOD1 Rats and Amends the Course of ALS
Source: Stem Cells. 2014 Nov 26;32(12):3163–72. doi: 10.1002/stem.1812 (PMC4321196; doi:10.1002/stem.1812)
Supplement: Supplementary file 6 — Supplementary [file stem0032-3163-SD6.doc]

**SUPPLEMENT FIGURES.**

**Figure S1.**

MSCs that were used for intrathecal transplantation expressed the following human surface markers profile evaluated using FACS (A). MSCs were also able to differentiate toward the osteogenic (B), adipogenic (C) and chondrogenic (D) phenotypes and were positive for vimentin (E) marker. (Scale bars: B-D= 200µm and E= 50µm).

**Figure S2.**

Representative immunofluorescence staining for versican in the ventral horns of wild-type (A-D), end-stage SOD1 MSC-treated (E-H) and symptomatic SOD1 sham-treated (I-L) rats. Neurons are visualized with anti-NeuN antibody. Scale bars = 50µm.

**Figure S3.**

Representative immunofluorescence staining for aggrecan in the ventral horns of wild-type (A-D), end-stage SOD1 MSC-treated (E-H) and symptomatic SOD1 sham-treated (I-L) rats. Neurons are visualized using the anti-NeuN antibody. Scale bars = 50µm.

**Figure S4.**

Representative immunofluorescence staining for link protein-1(hapln-1) in the ventral horns of wild-type (A-D), end-stage SOD1 MSC-treated (E-H) and symptomatic SOD1 sham-treated (I-L) rats. Neurons are visualized using the anti-NeuN antibody. Scale bars = 50µm.

**Figure S5.**

Representative immunofluorescence staining for phosphacan (3F8) in the ventral horns of wild-type (A-D), end-stage SOD1 MSC-treated (E-H) and symptomatic SOD1 sham-treated (I-L) rats. Neurons are visualized using the anti-NeuN antibody. Scale bars = 50µm.
