# Supplementary material for: Intrathecal Delivery of Mesenchymal Stromal Cells Protects the Structure of Altered Perineuronal Nets in SOD1 Rats and Amends the Course of ALS
Source: Stem Cells. 2014 Nov 26;32(12):3163–72. doi: 10.1002/stem.1812 (PMC4321196; doi:10.1002/stem.1812)
Supplement: Supplementary file 7 — Supplementary [file stem0032-3163-SD7.docx]

## METHODS SI

## *Animals*

As an animal model of familial amyotrophic lateral sclerosis, we used transgenic male Sprague Dawley rats (SD-Tg SOD1(G93A)L26H) that overexpress human SOD1 and carry the Gly-93-Ala mutation (Taconic, USA). The rats were bred in our animal facility from breeding couples. All animals were housed under standard laboratory conditions: a 12:12 h dark:light cycle, at 23ºC room temperature, two rats per cage, with food and water supplied *ad libitum*. This study was performed in accordance with the European Communities Council Directive of 24 November 1986 (86/609/EEC) regarding the use of animals in research, and was approved by the Ethics Committee of the Institute of Experimental Medicine, AS CR.

## *Behavioral testing*

To evaluate the beginning, progression and end-stage of the disease, the same person tested motor functions and measured body weights of the animals at the same time of day and on the same day of the week (unless more frequent testing was required). For this purpose we trained rats to undergo a combination of previously described motor tests: the grip strength, BBB and thirty seconds tests [[1](#_ENREF_1)]. Brief descriptions of the used tests are as follows:

### Grip strength test

The grip strength test is one of the most sensitive tests for determining the onset and end-stage of ALS, as well as to study the effect of cell therapy on the course of the disease. The animal was allowed to grasp a metal grid and then was pulled backwards, in the horizontal plane, by the examiner. The maximum force generated by the animal just before it lost its grip was recorded in grams by a grip strength meter (Grip Strength Meter BSGT2S, Harvard Apparatus, Holliston, MA, USA).

### BBB test

The animal’s motor activity was evaluated by the BBB test, a unified scale developed by Basso, Beattie and Bresnahan to grade spinal cord injury [[2](#_ENREF_2)]. The animal’s trunk stability, forelimb-hindlimb co-ordination during gait, limb advancement, paw placement, etc. were graded using a 21 point scale, with 21 indicating a full range of movement (healthy rat) and 0 the total absence of limb movements.

### Thirty seconds test

The end stage of ALS was confirmed by the “thirty seconds test” in an additional to the grip strength test, BBB test and body weight measurements. The animals were placed on their side and the time spent to position themselves back into their original standing posture was measured. When the rats were unable to right themselves within 30 seconds, their condition was considered to be in the end stage of the disease, and they were consequently sacrificed.

The beginning of the disease was established individually for each rat when two out of three tested parameters started to decline: BBB score dropped from 21 to 17–16, grip strength decreased by more than 200 g compared to the individual baseline established for each animal, or the animal started to lose body weight. To confirm diagnosis, rats experiencing the first symptoms of motor deficiency (between 170-180 days of age) were observed for five to seven days. When confirmed, animals were randomly divided into groups and considered for either mesenchymal stem cell (MSC) application (n=11) or vehicle-injection (n=9); wild type (WT) littermates were used as MSC-treated (n=5) and vehicle-injected (n=5) controls, as well as in a survival-biodistribution study with rats MSC^GFP+^ cells (n=6). In total 36 rats were used in the current trial.

Rats were considered to be in the terminal stage of ALS when they met two out of the following three criteria in any combination: 75% decline in motor activity (BBB score drop from 21 to 5), a decline in grip strength of 75% (this generally corresponded to a grip strength decrease from 2200 g to 500 g), a 30% decline in body weight (from 450 g to 320 g) and additionally were unable to right themselves when placed on their side for 30 seconds. For ethical reasons animals were sacrificed after reaching a terminal stage of the disease.

## *Electrophysiogical measurements*

Electrophysiological measurements were performed at the age of eight weeks (to obtain baseline data), two weeks before the expected manifestation of ALS symptoms and every subsequent week until the end-stage of the disease. An electromyography (EMG) stimulator was used to perform nerve conduction measurements and motor unit number estimation (MUNE); which refers to a group of techniques used to estimate the number of intact motor units, potentially serving as indirect evidence of ALS progression. In human medicine it is used as an additional method to diagnose several diseases of the motor system. The SOD1 animals used for current study (MSC-treated [n=6] and vehicle-treated [n=6]), were anesthetized by ketamine (Narkamon, i.p., 100-200 mg per kg) and were tested in a prone position using a standard EMG stimulator (Medelec Synergy, Oxford Instruments).

### a) Motor nerve conduction velocity test

The sciatic nerve was stimulated using monopolar needle electrodes (Teca Elite), with the cathode placed at the proximal part of the thigh and the anode inserted subcutaneously about 1 cm proximal to the cathode. The nerve was stimulated by means of single electrical pulses of 0.1 ms duration. The compound muscle action potential (CMAP) was recorded from the gastrocnemius muscle.

### b) Motor unit number estimation

MUNE refers to a group of techniques used to estimate the number of intact motor units that innervate a single muscle. The number of functional motor units corresponds to the number of spinal cord MNs, thus the estimation of motor unit number in the muscle can provide indirect evidence of ALS progression. The single electrical pulse settings for MUNE were the same as those used in the motor nerve conduction tests. We used a protocol involving an incremental technique according to McComas [[3](#_ENREF_3)]. Starting from sub-threshold intensity, the sciatic nerve was stimulated with single pulses of gradually increasing intensity until the first response appeared, representing the first motor unit recruited. With the next stimuli, quantal increases in the response were recorded. Increments larger than 50 µV were considered to be the recruitment of an additional motor unit. The amplitude of a single motor unit was calculated as the mean of 10-15 consistent increases. The estimated number of motor units was determined from the equation: MUNE=CMAP maximal amplitude/mean amplitude of single motor unit action potentials.

## *Human bone marrow mesenchymal stromal cell cultivation*

The human bone marrow MSCs used in our experiments were prepared under good manufacturing practice (GMP) conditions and provided by BioInova, s. r. o., a subsidiary of the Institute of Experimental Medicine ASCR, v.v.i. After obtaining the donor’s informed consent, bone marrow samples were collected by aspirating approximately 30-50 ml from the iliac crest of the patient. The mononuclear fraction containing MSCs was collected using Ficoll density-gradient centrifugation and was then transferred into a sterile tube (Ficoll-Paque Plus; GE Healthcare Bio-Sciences AB, Uppsalla, Sweden). The isolation of MSC-fraction was enabled by the selective adherence of MSCs to plastic surfaces; nonadherent cells were removed after 48 hours by replacing the medium. Adherent cells were cultivated at 37°C in a humidified atmosphere containing 5% CO_2_, and the medium was changed twice a week. Cell isolation and expansion were performed using α-minimal essential medium (α-MEM, Gibco-Invitrogen, Carlsbad, CA, USA). MSCs from passage three were used for intrathecal application and characterized for mesenchymal features using fluorescence-activated cell sorting (FACS) surface marker profiles and the cells’ ability to differentiate into adipogenic, osteogenic and chondrogenic phenotypes [[4](#_ENREF_4), [5](#_ENREF_5)]. All MSCs used in the experiments were isolated from one human donor and thus all MSCs-treated rats were treated with the cells cultivated and prepared for application from the same passage.

## *Rat bone marrow mesenchymal stromal cell cultivation*

We also used rat MSCs (5×10^5^ cells/50µl) that expressed green fluorescent protein (MSC^GFP+^) for evaluation of survival-biodistribution after intrathecal cell delivery into WT rats. Rat MSC^GFP+^ were cultured as previously described by our group [[1](#_ENREF_1)].

## *Intrathecal cell application*

To avoid the possibility of graft versus host disease (GVHD), all animals before MSCs/vehicle application were immunosuppressed by a combination of Sandimmun (10 mg/kg; Novartis Pharama AG, Switzerland), Immuran (4 mg/kg; GlaxoSmithKline, USA) and Solu-Medrol (2 mg/kg; Pfizer, Belgium) [[6](#_ENREF_6)]. Animals were anesthetized prior to any manipulation that could cause pain; a general anesthesia was conducted with 3% vapor inhalation of Isoflurane (Forane, Abbot Laboratories, Ltd., Queenborough, Great Britain). When full anesthesia was achieved, the rat was placed into a stereotaxic apparatus and further anesthetized with 3 percent vapor inhalation of Isoflurane. All surgeries were performed using an OPMI-1 surgical microscope (Carl Zeiss, Oberkochen, Germany) at 15-25x magnification under aseptic conditions. After shaving the fur and cleaning the skin above the base of the skull, a 1.5 centimeter cut was made. The muscles were retracted to expose the *dura* overlaying the cistern magna. The subdural space was accessed via a small incision of the *dura* and confirmed by the return of CSF. Visibility was maintained by using a sparing blot, taking care not to remove the CSF. In order to minimize possible nerve damage, a fine catheter (ALZET, cat. No 0007741) was connected via a gauge to a 50 µl Hamilton syringe (Hamilton Bonaduz AG, Bonaduz, Switzerland) and filled with a cell suspension (in total 5×10^5^ cells in 50µl of vehicle). The catheter was gently inserted into the aperture to the *cisterna magna* with the tip advanced backward to the C8-Th1 level of the spinal cord. Subsequently, the cell suspension/vehicle (total volume 50µl) was injected intrathecally at a speed of 25 μl/min using a Nano-Injector (Stoelting Co.). After the cell application the catheter was removed and the wound was sutured in anatomical layers.

Following the same protocol MSC^GFP+^ (5×10^5^ cells/50µl) were injected for the evaluation of survival and biodistribution of grafted cells in different organs of healthy WT recipient rats (n=6). Fourteen days after cell transplantation we collected the spinal cord, brain, lungs, spleen, liver, and CSF for the histological evaluation.

## *Postoperative care*

After the surgery all animals received an intramuscular (0.3ml) bolus injection of Ampicillin (Biotika) and a subcutaneous (0.1ml) injection of Rimadyl (Pfizer, Puurs, Belgium). All animals were immunosuppressed by a combination of drugs: Sandimmun, Immuran and Solu-Medrol lifelong (aforementioned).

## *Immunohistochemistry*

Spinal cords (n=36) were dissected and post-fixed in 4% paraformaldehyde solution, cryoprotected in a sucrose gradient (10-30% in 0.2M phosphate buffer) and cut into three parts: one for longitudinal (from T1 to T10) and two for transverse sections (C1-C8 and T11-L4). Longitudinal sections were used to study the fate of the transplanted cells (IHC staining for human MTC02 and HuNu markers), whereas transversal sections were used for quantitative evaluation (MN numbers, PNNs analysis and terminal deoxynucleotidyltransferase (TdT)-mediated dUTPbiotin nick-end labeling assay [TUNEL-assay]). Sections were blocked with a 3% goat serum and 3% bovine serum albumin in Tris buffer with 0.2% Triton-X100 (Sigma-Aldrich), followed by incubation with primary and appropriate secondary antibodies. PNNs were visualized by biotinylated Wisteria floribunda agglutinin ([WFA] 20μg/ml, Sigma-Aldrich) labeled with streptavidin conjugated to Alexa488 (Molecular probes, 1:100). The following primary antibodies were used in the study: mouse anti-NF200 (Sigma, 1:200), rabbit anti-NeuN (Millipore, 1:200), mouse SMI-32 (Covance, 1:1000), mouse anti-MTC02 (Abcam, 1:200), rabbit anti-versican (Chemicon, 1:500), rabbit anti-aggrecan (Chemicon, 1:500), mouse anti-phosphacan (DSHB, 1:100), and goat anti-hapln1 (R&D Systems, 1:100). To visualize the cell nuclei, sections were stained with 4′6-diamidino-2-phenylindole dichlorhydrate (DAPI), after washing with PBS, and mounted with Aqua-Poly/Mount (Polysciences, Warrington, PA, USA). The sections were evaluated using a Zeiss LSM 5 DUO (Zeiss, Oberkochen, Germany) confocal microscope equipped with an Ar/HeNe laser.

For quantifying the fluorescent intensity of TUNEL, WFA and PNN-component (versican, aggrecan, link protein-1 [hapln1] and phosphacan) staining, images from an optical field of 315x315μm were taken using a Zeiss LSM 5 DUO confocal microscope and with 20x objective. Following this, the optical densities (grey scale levels of the corresponding pixels of the pre-processed image) along with the surface area were determined by means of Axio Vision4 software from the outline of both ventral horns, in at least five slices (taken with an interval of 100µm between each slice) from the thoracic and lumbar levels of the spinal cord. The background optical density, calculated from spinal cord sections processed without the addition of a primary antibody, was subtracted. The values reported for the thoracic and lumbar levels are the group means (SOD1+MSCs n=11, SOD1+DMEM n=9, WT+MSCs n=5 and WT+DMEM n=5) of the average fluorescence intensity from a single animal.

## *Quantitation of motoneuron numbers in the spinal ventral horns*

The fluorescent intensity and MN number were quantified at the cervical and lumbar spinal cord levels. We used an unbiased stereological method on serial sections in both ventral horns in at least five sections (twenty micrometer thick, taken with an interval of 100µm) using AxioVision4 software, as described previously [[1](#_ENREF_1)]. Neurons were visualized using rabbit anti-NeuN primary antibody and Alexa Fluor 594 conjugated anti-rabbit IgG secondary antibody. Images of the left and right ventral horns were recorded by means of a Zeiss Axio Observer microscope using a 20x objective and Axio Vision4 software (Carl Zeiss Vision GmbH, Germany). The background optical density, calculated by omitting the primary antibody, was subtracted. The values reported are the group means of the average fluorescence intensity from a single animal.

## *Gene expression*

The total amount of mRNA was extracted from the fixed spinal cords during the terminal stage of SOD1 rats (vehicle-treated [n=4], MSC-treated [n=5]) and healthy WT littermates of the same age (n=5) using the High Pure RNA Paraffin Kit (Cat. No. 03270289001; Roche, Germany) following the manufacturer’s instructions. The concentration of the total amount of RNA was measured using a Nano Drop Spectrophotometer (Nano Drop Technologies, USA). To perform RT-qPCR we used reagents and kits purchased from Applied Biosystems (Foster City, CA, USA). First strand cDNA was synthesized in duplicate from 10ng of mRNA (20 µl reaction volume) by means of a High Capacity cDNA Reverse Transcription Kit (Cat. No.4368813). The cDNA product was preamplified using TaqMan PreAmp Master Mix (Cat. No.4384267; Applied Biosystems).

Preamplification products were diluted 20 times in Tris-EDTA (TE) buffer as recommended by the manufacturer; and subsequently were used to quantitatively compare the gene expression of the target CSPGs (*Brevican, Versican, Aggrecan, Tenascin-R, Neurocan And Hapln1*). The RT-qPCR was carried out in a final volume of 10 μl. The amount of the PCR product was determined using the StepOnePlus^TM^ Real Time PCR System (Applied Biosystems), TaqMan Gene Expression Master Mix (Cat. No. 4369510) and TaqMan Gene Expression Assays (Cat. No.4331182) for *rattus norvegicus* (assay/gene name): Rn00573424/*Aggrecan*/, Rn00581331_m1/*Neurocan*/, Rn00563814_m1/*Brevican*/, Rn00564869_m1/*Tenascin-R*/, Rn01493755/*Versican*/ and Rn00569884_m1/*Hapln1*/. Thermal profiles were set as recommended by the manufacturer. All results were obtained using the integrated StepOne^TM^ Software (version 2.3) and further evaluated using the ΔΔΔCq method relative to two appropriate endogenous reference genes selected by NormFinder: *Brevican* (Rn00563814_m1) and *beta-Actin* (Rn00667869_m1). Gene expression was normalized against healthy WT littermates of the same age and compared between the groups. *Brevican* was evaluated using only the *beta-Actin* reference gene (Rn00667869_m1) using the ΔΔCq method. Finally, all data were recalculated to relative quantities and transformed to a log2 scale according to the MIQE (Minimum Information for Publication of Quantitative Real-Time PCR Experiments) recommendations [[7](#_ENREF_7)].

## Cytometric bead-based assay

A FlowCytomix™ Multiple Analyte Detection System (eBioscience, San Diego, CA, USA, formerly BenderMed, Austria) was used to quantify the cytokine levels in the cerebrospinal fluid (CSF) of SOD1^G93A^ rats, age-matched sham-treated littermates and WT animals at the terminal stage of the disease, prior to sacrificing the animals. The bead-based assay follows the same principle as a sandwich immunoassay and allows a simultaneous detection of up to 20 cytokine analytes in one sample. Flow cytometry is used to differentiate bead populations according to bead size and fluorescent signature. For our experiments, we used a rat cytokine 6plex FlowCytomix Kit (BMS825/3FF and a rat FlowCytomix Basic Kit (BMS8441FF). This method enables the quantitative detection of rat GM-CSF, IFN-γ, IL-1α, IL-4, MCP-1 and TNF-α. We prepared different standards including, a standard mixture, a bead mixture, a biotin-conjugate mixture and a streptavidim-PE solution according to the manufacturer‘s recommendation and in a volume sufficient for the number of samples. All of the samples (25 µl), standard mixture dilutions, blank and setup tubes were incubated with the bead mixture (25 µl) as well as the biotin-conjugate mixture (50 µl) for 2 hours, and then washed twice with assay buffer. Streptavidin-PE (50 µl) was added to all of the tubes and incubated for 1 hour. Samples were then washed twice in assay buffer, diluted in 500 µl of assay buffer and analyzed using a BD FACSAria^TM^ flow cytometer. The obtained raw data files were then assessed using FlowCytomix™ Pro 3.0 Software and transformed into absolute values in picograms per milliliter of a used sample. All samples were analyzed in duplicate.

## *Statistical Analysis*

All numeric data are presented as mean values and assessed with a Student’s t-test (Sigma-Plot 9.0); p<0.05 was considered significant, p<0.01 was considered very significant and p<0.001 was considered extremely significant. Group differences are presented as mean ± standard error of the mean (S.E.M.), and the Kaplan-Meier method (Origin 7.3) was used to determine the difference in survival rate between the groups of MSC- and sham-treated animals.

**REFERENCES**

1. Forostyak S, Jendelova P, Kapcalova M et al. Mesenchymal stromal cells prolong the lifespan in a rat model of amyotrophic lateral sclerosis. **Cytotherapy***.* 2011;13:1036-1046.

2. Basso DM, Beattie MS, Bresnahan JC. A sensitive and reliable locomotor rating scale for open field testing in rats. **Journal of neurotrauma***.* 1995;12:1-21.

3. McComas AJ. Motor unit estimation: anxieties and achievements. **Muscle Nerve***.* 1995;18:369-379.

4. Turnovcova K, Ruzickova K, Vanecek V et al. Properties and growth of human bone marrow mesenchymal stromal cells cultivated in different media. **Cytotherapy***.* 2009;11:874-885.

5. Dominici M, Le Blanc K, Mueller I et al. Minimal criteria for defining multipotent mesenchymal stromal cells. The International Society for Cellular Therapy position statement. **Cytotherapy***.* 2006;8:315-317.

6. Kozubenko N, Turnovcova K, Kapcalova M et al. Analysis of in vitro and in vivo characteristics of human embryonic stem cell-derived neural precursors. **Cell transplantation***.* 2010;19:471-486.

7. Bustin SA, Benes V, Garson JA et al. The MIQE guidelines: minimum information for publication of quantitative real-time PCR experiments. **Clin Chem***.* 2009;55:611-622.
